# Psychological impact of providing women with personalised 10-year breast cancer risk estimates

**DOI:** 10.1038/s41416-018-0069-y

**Published:** 2018-05-08

**Authors:** David P. French, Jake Southworth, Anthony Howell, Michelle Harvie, Paula Stavrinos, Donna Watterson, Sarah Sampson, D. Gareth Evans, Louise S. Donnelly

**Affiliations:** 1 Manchester Centre of Health Psychology, School of Health Sciences, University of Manchester, Manchester, M13 9PL UK; 2 Prevent Breast Cancer Centre and Nightingale Breast Screening Centre, University Hospital of South Manchester, Manchester, M23 9LT UK; 3 Manchester Breast Centre, Manchester Cancer Research Centre, University of Manchester, Manchester, M20 4BX UK; 4 The Christie NHS Foundation Trust, Manchester, M20 4BX UK; 5 Department of Genomic Medicine, Institute of Human Development, Manchester Academic Health Science Centre (MAHSC), Central Manchester University Hospitals NHS Foundation Trust, Manchester, M13 9WL UK; 6 Division of Evolution and Genomic Sciences, Faculty of Biology, Medicine and Health, University Manchester, Manchester, M13 9NT UK

**Keywords:** Human behaviour, Population screening

## Abstract

**Background:**

The Predicting Risk of Cancer at Screening (PROCAS) study estimated 10-year breast cancer risk for 53,596 women attending NHS Breast Screening Programme. The present study, nested within the PROCAS study, aimed to assess the psychological impact of receiving breast cancer risk estimates, based on: (a) the Tyrer–Cuzick (T-C) algorithm including breast density or (b) T-C including breast density plus single-nucleotide polymorphisms (SNPs), versus (c) comparison women awaiting results.

**Methods:**

A sample of 2138 women from the PROCAS study was stratified by testing groups: T-C only, T-C(+SNPs) and comparison women; and by 10-year risk estimates received: 'moderate' (5–7.99%), 'average' (2–4.99%) or 'below average' (<1.99%) risk. Postal questionnaires were returned by 765 (36%) women.

**Results:**

Overall state anxiety and cancer worry were low, and similar for women in T-C only and T-C(+SNPs) groups. Women in both T-C only and T-C(+SNPs) groups showed lower-state anxiety but slightly higher cancer worry than comparison women awaiting results. Risk information had no consistent effects on intentions to change behaviour. Most women were satisfied with information provided. There was considerable variation in understanding.

**Conclusions:**

No major harms of providing women with 10-year breast cancer risk estimates were detected. Research to establish the feasibility of risk-stratified breast screening is warranted.

## Introduction

Every year in the United Kingdom, ~52,200 women develop breast cancer and as a result, 11,400 will die.^1^ The National Health Service Breast Screening Programme (NHSBSP) aims to provide early detection and thereby reduces mortality from breast cancer.^2^ However, it has been argued that the NHSBSP causes harm due to false-positive test results and overdiagnosis, although these appear not to outweigh the benefits in terms of lives saved.^2^

One possible means of improving the balance of harms and benefits of screening is to develop risk-stratified screening, which is increasingly being considered internationally.^3^ The National Institute for Health and Care Excellence (NICE) recommended in 2013 that women at high risk of breast cancer should have more frequent screening by mammography (annual between 40 and 60 years) and be offered chemoprevention therapy (tamoxifen or raloxifene).^4^ However, these NICE guidelines cannot currently be implemented with women undergoing NHSBSP screening as women who attend are not currently assessed for their breast cancer risk.

To make risk stratification possible and for consequent benefits to women to be realised, a formalised approach to identifying and communicating women’s breast cancer risk is required. In line with this, the Predicting Risk of Cancer at Screening (PROCAS) study^5^ found that it was possible to accurately estimate the individual breast cancer risk of a population sample of women aged 46–73 years in Greater Manchester, England using the Tyrer–Cuzick (T-C) model.^6^ The T-C model was developed to estimate breast cancer risk using up to three sources of information: (a) self-reports, e.g., of family history, parity, BMI, height, age at menarche/menopause/at first live birth, HRT use, (b) breast density, obtained from mammography and (c) genetic information, i.e., single-nucleotide polymorphisms (SNPs) derived from saliva. Importantly, the additional cancers identified in higher-risk women were more likely to be stage 2 and above and women in these groups were therefore most likely to benefit from earlier detection.^6, 7^

Before a service that provides breast cancer risk estimates could be implemented, or tested in an effectiveness study, it is important to establish the likely harms and benefits of providing women with their personalised breast cancer risk. Potential harms of breast screening include overdiagnosis, invasive testing and incidental findings.^2^ A key set of harms and benefits to consider are the emotional, cognitive and behavioural impacts of receiving personalised breast cancer risk estimates^8^ that have been considered in depth for breast cancer screening.^9^ These include undue anxiety and worry about cancer in women informed of high risk. The main potential benefits of breast cancer risk feedback to women would consist of increased knowledge allowing more informed choices regarding prevention options (e.g., chemoprevention), as well as potential changes in risk reducing behaviours such as increased physical activity, and reduced alcohol consumption.

The main aim of the present research is to assess the psychological impact of receiving personalised estimates of breast cancer on women who participated in the PROCAS study. Specifically, this manuscript examines the psychological impact of receiving risk estimates based on: (a) the T-C model incorporating breast density information versus, (b) the T-C model including breast density information, and incorporating genetic information from SNPs extracted from saliva, with (c) comparison women also from the larger PROCAS study awaiting personalised risk information. For the T-C and T-C(+SNPs) groups, the impact of levels of risk estimates was also compared for three groups of women at 'moderate-risk' (10-year risk 5–7.99%), 'average risk' (10-year risk 2–4.99%) and 'below average risk' (10-year risk <2%).

## Materials and methods

### Design

The present research was approved by Liverpool East NHS Research Ethics Committee. It was a natural experiment of women from the larger PROCAS study, and employed a 3×3 between-subjects design, with each woman being allocated to one testing group and receiving one of three categorical risk results (shown in Table 1). Allocation to experimental condition did not employ randomisation. SNP testing was introduced after the PROCAS study had already begun, and SNP testing was carried out at sites where it was logistically easier to collect the data, e.g., proximity to study team and facilities for saliva testing. There were three testing groups:

- Women who received risk estimates based on the T-C algorithm, based on self-completed questionnaires and incorporating information on mammographic density.
- Women who received risk estimates based on the T-C algorithm, based on not only self-completed questionnaires and mammographic density, but also genetic information derived from SNPs.
- A comparison group of women who would receive their risk estimates in the near future, but were asked to complete questionnaires for the present study before receiving their risk estimates. Study team members were aware of estimated 10-year breast cancer risk of women in this group.

**Table 1:** Experimental design showing 3×3 experimental groups, with number of women recruited per experimental group

Risk result categories	Risk testing group		
	Risk estimation from Tyrer–Cuzick (including mammographic density) plus SNPs	Risk estimation from Tyrer–Cuzick (including mammographic density) only	Comparison women awaiting risk estimation
Moderate risk (5–7.99%)	87	72	99
Average risk (2–4.99%)	74	66	100
Below average risk (less than 2%)	110	59	98

Further, within each of these three groups, the risk of participating women was estimated to be within one of three categories: (a) 'moderate-risk' (between 5 and 7.99% risk over next 10 years), (b) 'average risk' (between 2 and 4.99% risk) and (c) 'below average risk' (less than 2% risk). Women identified as being at high risk, i.e., 8% or higher were excluded, as they had already received offers of individualised risk consultations by telephone or face-to-face with consultants.

### Participants

A total of 131,373 women in Greater Manchester who were invited for routine breast screening during 2009 and 2014 were also asked to participate in the overall PROCAS project, via invitation letter alongside their NHSBSP. In line with usual practice in England, women aged 50–70 years were invited, as well as women invited as part of the AgeX trial, who were 47–59 and 71–73 years (NCT01081288). Of women who attended for screening, a total of 53,596 women (40.8%) consented to the research when they attended their screening appointment, at designated sites within hospitals or in large vans in locations to cover populations that are not within easy reach of the hospital screening sites. Those women who consented then completed a two-page questionnaire to assess their personalised risk of breast cancer. Of these 53,596 women, 51,011 (95.2%) had requested receiving this breast cancer risk estimate in due course. The age of the PROCAS sample was between 47 and 73 years and the majority (90.9%) reported their ethnicity as 'white'.

In the overall PROCAS study, there were 3.2% of women identified as being at high risk, 10.3% of women identified as being at moderate risk, 59.3% of women were identified as being at average risk, and 27.2% of women were identified as being at below average risk.

### Procedure

Women in the present study were selected from the overall PROCAS study on the basis of being scheduled to receive their estimated breast cancer risk during the study period of November 2015 to August 2016. As the main PROCAS study had the central objective of evaluating the risk model, women who were invited to participate in the present study were expecting a considerable delay between being tested and receiving their results.

Women in the T-C, or T-C(+SNPs) groups who were included in the present study were recruited to the study and had risk assessment between May 2012 and July 2013; women in the comparison group were recruited and had risk assessment between September 2013 and March 2015. Women in the (a) T-C and (b) T-C(+SNPs) groups were posted letters containing risk estimates and explanatory leaflets between November 2015 and December 2015. Women in these groups were also posted questionnaires for the present study between January 2016 and April 2016, and women in the (c) comparison group were posted questionnaires for the present study between February 2016 and August 2016. Non-responders were posted reminders after 4 weeks.

The risk estimates were accompanied by explanatory leaflets that provided information on what their result meant, what they could do to reduce their risk and advice on detecting symptoms of breast cancer. The leaflets and letters were co-designed in line with guidance,^10^ and involved 'think aloud' interviews followed by semi-structured interviews with 37 women. Risk estimates were fed back in categories, following service user input into how best to communicate this information and what labels should be used to best convey personal risk (Evans et al.)^5^. No women in the present study received face-to-face consultations: these were only received by women at 'high risk' who were not included in the present study.

All participants were sent a participant information sheet for the present study, consent form, questionnaire and pre-paid envelope. Those women who did not respond within 4 weeks were sent a reminder and a further reminder if they still had not replied within a further 4 weeks.

### Measures

All participants were asked to complete the following measures:

State anxiety was assessed using the six-item short form^11^ of the Spielberger State-trait Anxiety Inventory,^12^ with participants indicated for six emotion adjectives (e.g., 'upset') their present feelings by selecting one of the following response options 'not at all', 'somewhat', 'moderately' and 'very much'; *α* = 0.86.

Breast cancer worry was assessed using the Lerman Cancer Worry Scale,^13^ consisting of six statements such as: 'how often have you thought about your chances of getting cancer'. Participants endorsed one of the following response options 'never', 'rarely', 'sometimes' and 'almost all the time'; *α* = 0.87.

Perceived relative risk of developing breast cancer was assessed using a single item that asked women to rate their risk of developing breast cancer in the next 10 years, compared with other women of their age, from 'much lower', 'a bit lower', 'about the same', 'a bit higher' and 'much higher'.^14^

Intentions to change five health-related behaviours related to breast cancer prevention were assessed using five-point scales adapted from previously published items:^15^ follow a calorie-controlled diet, be physically activity, attend next mammogram, drink less alcohol and discuss prevention options with their GP (response options 'strongly disagree', 'disagree', 'neither agree nor disagree', 'agree', 'strongly agree'). Intention to take up chemoprevention was assessed only for participants in the T-C, or T-C(+SNPs) groups.

Participants in the T-C, or T-C(+SNPs) groups but not the comparison group were also asked to complete the following measures.

Satisfaction with the information was assessed using four items (*α* = 0.86), adapted from a previously published scale,^16^ which asked women how clear they found the information, how confusing they found it, how well informed they feel about their breast cancer risk information and how satisfied they with the amount of information given. Response options were 'strongly disagree', 'disagree', 'disagree somewhat', 'undecided', 'somewhat agree', 'agree' and 'strongly agree'.

Understanding of test result was assessed by asking women to select the single option among the following 10 options provided in Table 2, which best described what their test result meant, adapted from a previously published item.^16^

**Table 2:** Demographic and clinical characteristics (mean [SD], % [*n*]) of testing groups, with some characteristics also broken down by test results

	Test groups			Test statistics	*P* values
	T-C plus SNPs	T-C only	Comparison		
	*n* = 271	*n* = 197	*n* = 297		
Age: mean (SD)	61.94 (6.91)	54.27 (4.19)	52.40 (2.91)	F (2762) = 281.8	***P*** < **0.001**
Moderate risk	62.24 (6.41)	55.10 (5.39)	52.90 (3.60)		
Average risk	61.43 (7.73)	53.73 (3.06)	51.91 (2.42)		
Below average risk	62.04 (6.75)	53.88 (3.44)	52.39 (2.51)		
BMI: mean (SD)	27.42 (5.58)	26.95 (4.81)	26.08 (4.59)	F (2732) = 5.0	***P*** = **0.007**
Moderate risk	28.38 (5.88)	26.69 (5.12)	25. 14 (3.71)		
Average risk	26.29 (4.39)	26.76 (4.96)	26.11 (4.96)		
Below average risk	27.57 (5.95)	26.95 (4.81)	26.94 (4.82)		
Ethnicity: % (*n*)^a,b^				*χ*^2^ (10) = 11.8	*P* = 0.30
White	93.3% (253)	90.9% (179)	92.3% (274)		
Asian or Asian British	0.7% (2)	2.0% (4)	1.0% (3)		
Black or Black British	0% (0)	1.5% (3)	1.7% (5)		
Mixed	1.1% (3)	1.0% (2)	0% (0)		
Other	2.6% (7)	2.5% (5)	3.4% (10)		
Not indicated	3.3% (6)	3.0% (6)	1.7% (5)		
IMD: mean (SD)	5.31 (2.68)	5.83 (3.02)	6.31 (2.88)	F (2762) = 8.7	***P*** < **0.001**
Moderate risk	5.83 (2.45)	6.07 (2.93)	6.56 (3.08)		
Average risk	5.65 (2.82)	5.92 (3.02)	6.69 (2.63)		
Below average risk	4.67 (2.65)	5.42 (3.13)	5.67 (2.84)		
Number of relatives affected by breast cancer: % (*n*)^a^				*χ*^2^ (4) = 12.0	***P*** = **0.017**
None	87.5% (237)	75.6% (149)	80.8% (240)		
One	11.8% (32)	23.4% (46)	18.9% (56)		
Two or more	0.7% (2)	1.0% (2)	0.3% (1)		
Mammography: % (*n*)^a^				*χ*^2^ (2) = 285.5	***P*** < **0.001**
First	30.3% (82)	(160)	93.9% (279)		
Repeat	69.7% (189)	18.8 % (37)	6.1% (18)		
Location: % (*n*)^a^				*χ*^2^ (10) = 226.7	***P*** < **0.001**
Trafford	35.8% (97)	26.4% (52)	18.5% (55)		
Withington	33.2% (90)	8.6% (17)	4.4% (13)		
Manchester city	10.7% (29)	7.6% (15)	7.1% (21)		
Oldham	11.1% (30)	18.3% (36)	14.1% (42)		
Salford	9.2% (25)	12.2% (24)	10.4% (31)		
Tameside	0% (0)	26.9% (53)	45.5% (135)		
Days from risk assessment to risk feedback: mean (SD)	1124.5 (95.9)	991.7 (80.9)	838.2 (67.3)	F (2762) = 869.4	***P*** < **0.001**
Moderate risk	1161.7 (81.1)	996.3 (84.6)	801.0 (100.2)		
Average risk	1068.8 (111.2)	994.7 (82.4)	853.3 (26.8)		
Below average risk	1132.6 (77.7)	982.7 (74.8)	860.4 (28.4)		

Values shown in bold are statistically significant (i.e. *P*<0.05 or lower.

^a^Chi-squared test

^b^Comparison of “white” vs “non-white” *χ*^2^ (1) = 0.99, *P* = 0.6

Demographic and clinical information was obtained from the PROCAS questionnaire that was used to estimate breast cancer risk, and information that was routinely collected by the NHSBSP. The index of multiple deprivation was obtained from participant postcode, and rates the area deprivation in deciles from 1 (most deprived) to 10 (least deprived).^17^

### Data analysis

To assess demographic and clinical differences in continuous variables between testing groups, ANOVAs were conducted with testing group as an independent fixed effect with three levels. Chi-squared analyses were conducted to assess differences between categorical demographic and clinical variables.

Effects on state anxiety, worry, risk perceptions and intentions to change each of five behaviours were examined independently. In each case, ANCOVAs were conducted with testing group and risk result group as independent fixed effects, and BMI, deprivation and age as covariates. For each variable, two analyses were conducted: (1) a comparison of participants in the T-C and T-C(+SNPs) groups, and (2) a comparison of participants in the combined T-C and T-C(+SNPs) groups vs participants in the comparison group. Two sets of sensitivity analyses were conducted, with these analyses repeated with additional covariates: (a) whether the initial mammogram was a first screen or repeat screen and (b) whether women had a subsequent mammogram scheduled before they completed the study questionnaire. These covariates did not make a significant contribution to the outcomes reported in Table 3 in either set of analyses, and had little effect on the results reported.

**Table 3:** Measures of anxiety, worry, perceived risk and overall satisfaction (mean [SD]), with statistical tests for differences by test groups, test results and their interaction

	Test results groups			Comparison T-C plus SNPs vs T-C only			Comparison intervention vs controls		
	T-C plus SNPs	T-C only	Control	Test group main effect	Test result group effect	Interaction (test group x test result)	Test group main effect	Test result group effect	Interaction (test group x test result)
	*n* = 252	*n* = 191	*n* = 292	Test statistics (with *p* values)	Test statistics (with *p* values)	Test statistic (with *p* values)	Test statistics (with *p* values)	Test statistics (with *p* values)	Test statistic (with *p* values)
State anxiety	9.45 (3.20)	10.15 (4.04)	11.29 (3.71)	F(1,434) = 1.0	**F(2,434)** **=** **14.9**	F(2,434) = 1.6	**F(1,726)** **=** **17.1**	**F(2,726)** **=** **7.3**	**F(2,726)** **=** **3.8**
				*P* = 0.312	***P*** **<** **0.001**	*P* = 0.205	***P*** **<** **0.001**	***P*** **=** **0.001**	***P*** **=** **0.024**
Moderate risk	10.87 (3.13)	10.98 (3.88)	11.50 (3.28)						
Average risk	8.95 (2.68)	10.42 (4.03)	11.08 (3.85)						
Below average risk	8.76 (3.27)	8.89 (3.97)	11.30 (3.96)						
Cancer worry	12.82 (3.26)	12.77 (2.96)	12.35 (3.21)	F(1,434) = 0.9	**F(2,434)** **=** **3.3**	F(2,434) = 1.2	**F(1,726)** **=** **3.9**	F(2,726) = 2.1	F(2,726) = 0.4
				*P* = 0.355	***P*** **=** **0.038**	*P* = 0.293	***P*** **=** **0.047**	*P* = 0.124	*P* = 0.702
Moderate risk	13.09 (2.90)	13.32 (2.99)	12.42 (3.08)						
Average risk	12.62 (3.30)	12.89 (2.97)	12.49 (2.88)						
Below average risk	12.75 (3.49)	12.03 (2.82)	12.15 (3.65)						
Comparative risk perceptions	2.17 (1.10)	2.83 (1.08)	2.99 (0.77)	F(1,433) = 0.8	**F(2,433)** **=** **352.5**	F(2,433) = 2.1	**F(1,725)** **=** **8.3**	**F(2,725)** **=** **170.2**	**F(2,725)** **=** **86.7**
				*P* = 0.364	***P*** **<** **0.001**	*P* = 0.127	***P*** = **0.004**	***P*** **<** **0.001**	***P*** **<** **0.001**
Moderate risk	3.84 (0.52)	3.86 (0.39)	3.08 (0.76)						
Average risk	3.77 (0.66)	2.85 (0.56)	3.11 (0.69)						
Below average risk	1.87 (0.88)	1.64 (0.80)	2.76 (0.82)						
Overall satisfaction with information	22.91 (4.14)	22.99 (4.14)		F(1,431) = 0.4 *P* = 0.544	**F(2,431)** **=** **9.2** ***P*** **<** **0.011**	F(2,431) = 2.6 *P* = 0.077			
Moderate risk	20.57 (4.77)	22.08 (4.15)							
Average risk	23.90 (3.41)	22.85 (4.73)							
Below average risk	23.99 (3.34)	24.21 (3.01)							

Values shown in bold are statistically significant (i.e. *P*<0.05 or lower)

Intentions to take chemoprevention and satisfaction with information were also examined using ANCOVAs as above, but only comparing participants in the T-C and T-C(+SNPs) groups.

## Results

Overall, 2138 women who were scheduled to receive feedback in 2015/2016 were sent questionnaires. Responses were received from 765 women (36% overall response rate). At least 200 questionnaires were sent to women in each of the nine experimental conditions.

### Matching of testing groups

The testing groups were not generally well matched (Table 4), with differences in a number of demographic and clinical variables. There was also variation in the number of days from being tested for the PROCAS study to being sent a risk estimate. It is important to note that, for most variables, the T-C(+SNPs) and comparison groups were most dissimilar, with the T-C group being intermediate. For some variables, such as age, women in the T-C group (mean age = 54.3 years) and comparison group (mean age = 52.4 years) were similar compared to the women in the T-C(+SNPs) group (mean age = 61.9 years). Women in the comparison group were, on average, younger, with lower BMI and least deprivation.

**Table 4:** Intentions to change behaviours (mean [SD]), with statistical tests for differences by test groups, test results and their interaction

Intentions to…	Test result groups			Comparison T-C plus SNPs vs T-C only			Comparison intervention vs controls		
	T-C plus SNPs	T-C only	Control	Test group main effect	Test result group effect	Interaction (test group x test result)	Test group main effect	Test result group effect	Interaction (test group x test result)
	*n* = 252	*n* = 189	*n* = 292	Test statistics (with *P* values)	Test statistics (with *P* values)	Test statistic (with *P* values)	Test statistics (with *P* values)	Test statistics (with *P* values)	Test statistic (with *P* values)
Follow a calorie-controlled diet	3.28 (0.90)	3.31 (0.97)	3.20 (0.99)	F(1,432) = 0.4	F(2,432) = 0.5	F(2,432) = 1.9	F(1,724) = 0.3	F(2,724) = 1.3	P(2,724) = 0.3
				*P* = 0.523	*P* = 0.582	*P* = 0.157	*P* = 0.563	*P* = 0.261	*P* = 0.733
Moderate risk	3.49 (0.81)	3.25 (1.00)	3.22 (1.00)						
Average risk	3.11 (0.89)	3.32 (0.92)	3.09 (0.98)						
Below average risk	3.24 (0.95)	3.36 (1.01)	3.28 (1.00)						
Be physically active	3.97 (0.79)	4.03 (0.73)	3.99 (0.84)	F(1,432) = 0.1	F(2,432) = 1.0	F(2432) = 0.8	F(1,724) = 2.1	F(2,724) = 1.0	F(2,724) = 0.6
				*P* = 0.719	*P* = 0.377	*P* = 0.471	*P* = 0.152	*P* = 0.351	*P* = 0.557
Moderate risk	3.97 (0.84)	4.12 (0.71)	4.03 (0.86)						
Average risk	3.94 (0.83)	4.05 (0.64)	3.93 (0.87)						
Below average risk	3.98 (0.73)	3.92 (0.82)	4.02 (0.79)						
Drink less alcohol	3.65 (0.90)	3.49 (0.89)	3.34 (0.88)	F(1,420) = 2.3	F(2,420) = 0.5	F(2,420) = 1.1	**F(1,706)** **=** **5.8**	F(2,706) = 0.4	F(2,706) = 0.0
				*P* = 0.133	*P* = 0.598	*P* = 0.350	***P*** **=** **0.016**	*P* = 0.659	*P* = 0.957
Moderate risk	3.80 (0.88)	3.46 (0.89)	3.36 (0.93)						
Average risk	3.58 (0.90)	3.48 (0.85)	3.32 (0.82)						
Below average risk	3.59 (0.93)	3.53 (0.94)	3.35 (0.91)						
Attend my next mammogram	4.76 (0.58)	4.67 (0.83)	4.78 (0.61)	**F(1,431)** **=** **3.9**	F(2,431) = 1.3	F(2,431) = 0.1	F(1,723) = 0.1	F(2,723) = 0.9	F(2,723) = 0.1
				***P*** **=** **0.048**	*P* = 0.264	*P* = 0.989	*P* = 0.729	*P* = 0.420	*P* = 0.851
Moderate risk	4.79 (0.50)	4.71 (0.79)	4.81 (0.64)						
Average risk	4.84 (0.37)	4.71 (0.65)	4.79 (0.56)						
Below average risk	4.70 (0.74)	4.58 (1.04)	4.74 (0.63)						
Discuss prevention options with my GP	2.87 (0.96)	2.82 (1.03)	2.84 (0.97)	F(1,432) = 0.0	F(2,432) = 2.8	F(2,432) = 0.4	F(1,724) = 0.8	F(2,724) = 0.9	F(2,724) = 2.3
				*P* = 0.939	*P* = 0.062	*P* = 0.678	*P* = 0.380	*P* = 0.425	*P* = 0.100
Moderate risk	3.01 (0.90)	2.97 (1.00)	2.76 (1.00)						
Average risk	2.71 (1.02)	2.77 (0.99)	2.92 (0.79)						
Below average risk	2.87 (0.95)	2.70 (1.10)	2.83 (1.10)						
Take a drug for prevention of breast cancer									
Moderate risk	2.54 (0.92)	2.45 (0.94)	3.48 (0.92)	F(1,136) = 0.0			**F(1,230)** **=** **58.4**		
				*P* = 0.986			***P*** **<** **0.001**		
Average risk			3.61 (0.99)						
Below average risk			3.74 (0.94)						

Values shown in bold are statistically significant (i.e. *P*<0.05 or lower)

### Psychological impact of receiving personalised risk estimates

There were no significant differences in state anxiety between women in the T-C and T-C(+SNPs) groups (Table 3). By contrast, women in the comparison group had higher anxiety levels than women in the T-C and T-C(+SNPs) groups. In both analyses, women with higher personal risk were more anxious (*P* < 0.001), although there was a significant moderator effect, where women in the comparison group had higher anxiety levels irrespective of personal risk levels.

When these analyses were repeated using the cutoff for anxiety disorder previously suggested,^12^ the pattern of results was highly similar. Of 763 women who completed the STAI, 111 (14.5%) exceeded this cutoff. This proportion was higher (*χ*^2^ = 11.28, df = 1, *N* = 763, *P* = 0.001) in 59/296 women in the comparison group (19.9%) relative to 52/412 women in the T-C and T-C(+SNPs) groups (12.5%). The proportions were not significantly different (higher (*χ*^2^ = 1.46, df = 1, *N* = 467, *P* = 0.144) between 26/197 women in the T-C only group (13.2%) and 26/270 women in the T-C(+SNPs) group (9.6%). In the T-C and T-C(+SNPs) groups, proportions of women exceeding the cutoff was related to risk estimate received (*χ*^2^ = 10.22, df = 2, *N* = 467, *P* = 0.006), with differences between women who received moderate (28/159 or 17.6%), average (11/140 or 7.9%) and below average (13/168 or 7.7%) risk estimates

There were no significant differences in cancer worry between women in the T-C and T-C(+SNPs) groups, although women with higher personal risk were more worried (see Table 3). Women in the comparison group reported less cancer worry than women in the T-C and T-C(+SNPs) groups.

There were no significant differences in perceived risk between women in the T-C and T-C(+SNPs) groups (Table 3). In this analysis, women who were told their personal risk was higher accordingly rated their personal risk as higher. In comparisons of women in the T-C and T-C(+SNPs) groups with women in the comparison group, women in the comparison group generally rated their personal risk as higher, and women who received higher personal risk estimates accordingly rated their personal risk as higher. In these later analyses, the results were subject to a significant interaction effect, where the association between personal risk group and perceived risk was much lower for women in the comparison group (who had not been informed of their personal risk level before they completed the questionnaire).

There was little evidence of differences between the T-C and T-C(+SNPs) groups in terms of satisfaction with information received (Table 3). Satisfaction was lower in response to higher-risk results than in response to lower risk results. This effect was modified by an interaction with average risk results associated with lower satisfaction for the T-C group and higher satisfaction with the T-C(+SNPs) group.

There were generally few effects of either testing group or test result group on intentions to change a variety of behaviours (Table 5).

**Table 5:** Percentage (*n*) of respondents in the T-C (+SNPs) and T-C only test result groups endorsing statements indicating understandings of the meaning of their latest test result, and their associated anxiety and concern (mean, SD)

Understanding of test result	T-C plus SNPs (*n* = 271)			T-C only (*n* = 197)		
	% (*n*)	Anxiety mean (SD)	Concern mean (SD)	% (*n*)	Anxiety mean (SD)	Concern mean (SD)
I am unlikely to develop breast cancer	24.0% (65)	9.23 (2.90)	12.62 (2.73)	29.9% (59)	9.59 (3.32)	12.76 (2.75)
I definitely do not have breast cancer	23.2% (63)	8.88 (3.11)	12.45 (3.72)	22.8% (45)	9.84 (4.56)	12.02 (2.58)
I am unlikely to have breast cancer	16.2% (44)	9.44 (3.08)	12.59 (3.29)	15.2% (30)	9.53 (3.70)	13.03 (3.12)
I am likely to develop breast cancer	8.5% (23)	12.06 (3.44)	14.65 (3.32)	13.7% (27)	11.85 (5.15)	14.04 (3.44)
I am very unlikely to develop breast cancer	11.8% (32)	7.44 (2.35)	11.77 (2.53)	8.1% (16)	9.81 (4.02)	12.25 (2.11)
This result does not tell me anything about my future likelihood of breast cancer	10.0% (27)	10.35 (2.85)	12.93 (3.11)	4.6% (9)	10.78 (2.86)	12.44 (4.22)
I do not know what my test result means	1.1% (3)	13.33 (5.13)	18.67 (4.62)	2.0% (4)	10.5 (3.42)	12.25 (2.22)
I am likely to have breast cancer	1.5% (4)	13 (5.10)	14.25 (2.99)	1.0% (2)	14 (2.83)	16 (5.66)
I am very likely to develop breast cancer	1.8% (5)	12.12 (3.80)	14.40 (5.41)	0.5% (1)	13	18
I have breast cancer	0.4% (1)	9	14	0.5% (1)	15	16
Missing	1.5% (4)	13.90 (2.04)	15.05 (1.38)	1.5% (3)	12 (1.00)	13.67 (1.15)

There was considerable variation in understanding of test results (see Table 2). Participants in the T-C and T-C(+SNPs) groups were asked to select one option from the 10 possible options that they felt best described what their result meant. Five response options were selected by at least 10% of participants, of which three were in line with the intended aim of the risk communications. These three options were 'I am unlikely to develop breast cancer' (26.5% of respondents), 'I am unlikely to have breast cancer' (15.8%) and 'I am very unlikely to develop breast cancer' (10.3%). By contrast, the other two options were at odds with the intended aim of communications: 'I definitely do not have breast cancer' (23.1%) and 'I am likely to develop breast cancer' (10.7%). Participants who selected this last option also had high state anxiety and cancer worry scores. This option was selected more often by women in the T-C group (13.7% or 27/197) than by women in the T-C(+SNPs) group (8.5% or 23/271). It is also notable that 10% (27/271) of women in the T-C(+SNPs) groups selected 'This result does not tell me anything about my future likelihood of breast cancer', in contrast to only 4.6% (9/197) of women in the T-C group.

## Discussion

There were very few differences on any measure between the T-C only and T-C(+SNPs) groups. Women in both the T-C and T-C(+SNPs) groups had lower-state anxiety levels than women in the comparison group who did not know their risk. Women with higher personal risk were more anxious in all groups, although there was a significant moderator effect, where women in the comparison group had higher anxiety levels irrespective of personal risk levels. Women in the T-C and T-C(+SNPs) groups had slightly higher cancer worry than women in the comparison group. Overall, state anxiety and cancer worry levels were low. There was little evidence that risk communication had effects on intentions to change any behaviour. Women were generally satisfied with information provided, and risk perceptions were in line with the information that had been communicated. When women in the T-C and T-C(+SNPs) groups were asked to select options that they felt best described what their result meant, there was considerable variation in understanding of test results with some misunderstandings apparent.

### Strengths and weaknesses

The present research has two features that make it highly innovative. First, the present research reports on the effects of communicating personalised breast cancer risk to women from the general breast screening population for the first time. Previous studies of the effects of communicating personalised risk across a range of conditions have focussed on highly selected samples in research settings.^18^ Further, studies of the effects of communicating genetic risk information have had an even narrower focus on clinical samples that were selected on the basis of existing high-risk previously detected by phenotypic markers.^19^ By contrast, in the present research, risk estimates were offered to a population sample of women who attended routine breast cancer screening. This makes the present findings much more informative about the likely population impact of risk communication in the context of risk-stratified screening.^8^

The present research compares risk estimates derived from phenotypic sources with risk estimates derived from both phenotypic and genotypic sources, to allow a comparison of how people respond to these two kinds of risk information. An innovative feature of this comparison is that in the present research, risk estimates using genotypic information were based on a large number of genetic markers that allowed more fine-grained estimation of risk.^5^ The use of a well-validated model incorporating genetic risk information stands in contrast to much research on the effects of communicating of genetic risk that is based on risk conferred by single allele mutations.^19^

A further strength of the present approach is that the approach taken to risk communication involved considerable user input. First, the decision to communicate risk estimates using categorical descriptors of risk was taken following considerable input from a patient-public involvement (PPI) user panel. Second, the letters and leaflets that were used to communicate risk estimates and the implications of this risk information were developed using several rounds of co-design with women who were drawn from the same population as women in the present research.

A major limitation of the present research is that the three testing groups of women were identified on the basis of convenience in communicating risk estimation and were not well matched on a variety of demographic, clinical and service-level variables. Although this lack of matching is far from ideal, there are good reasons to believe that it has not had a major biasing effect on the results reported. Importantly, for most demographic, clinical and service variables the T-C and comparison groups were similar, with the T-C(+SNPs) group being most different. For instance, women in the T-C group (mean age = 54.3 years) and comparison group (mean age = 52.4 years) were of similar age, but women in the T-C(+SNPs) group were considerably older (mean age = 61.9 years). By contrast, the T-C and T-C(+SNPs) groups showed a similar pattern of responding on the psychological impact variables, which were different from women’s responses in the comparison groups. Thus, it is implausible for the differences in demographic, clinical and service-level variables to be able to account for the results reported. There was no major seasonal variation or major trends in breast cancer screening attendance across England and the North-West of England where the study took place using data routinely reported by Cancer Research UK/ National Screening Committee.^5^

A second limitation of the present research lies in the long duration between consenting to receive risk estimates and receiving risk results, typically around three years (Table 4). This was an inevitable feature of the PROCAS study, which had a primary aim of validating the T-C algorithm and hence required some delay between risk estimation and breast cancers to be clinically detected.^5^ Nevertheless, the impact of receiving risk estimates three years after consenting to receive these may well have reduced the psychological impact of those estimates. It should be noted however, that questionnaires were completed around three-months following the receipt of risk estimates, so the impact on women receiving risk estimates was comparatively recent.

A thjrd limitation of the present research relates to the representativeness of the PROCAS sample, with 40% of women attending screening taking up the offer of risk estimation. As previously reported,^20^ deprivation scores were substantially lower in women who took up the offer of risk estimation compared to women who declined this offer. This is somewhat inevitable, as around 72% of eligible women currently attend breast screening,^21^ and of those, women had to consent to participate in the PROCAS study to be eligible for the present research. Based on this, it could be argued that participants were a group that were particularly keen to know their breast cancer risk estimate and this may have influenced the psychological impact of the information. This is possible, although given uptake rates in the NHS BSP and the requirement that people consent to research, there is little that could be done to ameliorate this problem. Further, if risk estimation were to be offered as part of routine NHS BSP, it would be expected that uptake would be socially patterned, so our results may mirror this. Finally, the present analyses controlled for deprivation, to ensure that deprivation did not confound comparisons between groups.

A final limitation is that there was only a 36% response rate to the questionnaires in the present study. Thus, it is possible that non-responders could have more or less anxiety and cancer worry than responders, with the results obtained concealing a substantial number of women who had higher levels of distress. Although the non-response rate was relatively similar between the groups, underlying differences and bias could exist in non-responders between the groups.

### What this study adds to existing literature

The present results show that women receiving personalised risk information have lower-state anxiety levels and only slightly higher cancer worry than women who were awaiting their risk estimates. It is important to note that overall, the levels of state anxiety and cancer-specific worry were low. The higher levels of state anxiety observed in the comparison group in the present study are consistent with the observation that uncertainty may provoke more anxiety than definitive bad news.^16^ That is, the levels of state anxiety in women in the comparison group are similar to those of women at moderate-risk in the other two testing groups, whereas women at average or below average risk in these two testing groups have lower anxiety levels. It should be noted however, that the present study found evidence that cancer-related worry was slightly higher in women who received risk estimates than in the comparison group. These findings are consistent with the observation that false-positive breast screening test results can result in slight elevations in cancer-related distress in the long term, even when general state anxiety shows no effects of such test results.^9^ Overall, mindful of the limitations of the evidence generated by the present study, these results are consistent with the hypothesis that providing women with breast cancer risk estimates does not produce large effects on anxiety or cancer worry.

The present study found no consistent effects of communicating personalised breast cancer risk on intentions to change lifestyle behaviour. Such a finding is consistent with a recent systematic review of systematic reviews of the effects of communicating personalised disease risk on lifestyle behaviour.^18^ This review found that there was little effect of personalised risk on behaviour, especially when risk information is communicated in numerical form, as opposed to using visual or other vivid means of feedback. These findings are also in line with a systematic review of the effects of communicating genetic risk information on behaviour, which found even clearer evidence that genetic risk information has little effects on behaviour.^19^ It is also notable that women who were told that they were at lower risk than other women did not show intentions to engage in unhealthier behaviour. This finding is in line with systematic reviews which show a general absence of effects on behaviour due to false reassurance from negative screening results.^22^

### Implications for practice

The major implication of the present research is that there is no compelling evidence that communicating personalised breast cancer risk estimates causes any major harms. There may be effects on cancer-related worry, but these effects were small. Further, there may be room to ameliorate such effects by improved communication of risk information. The ratings of perceived risk in the present study were consistent with the idea that women in the T-C and T-C(+SNPs) groups understood the risk information they had received, and satisfaction with information was generally high. However, there was evidence that satisfaction was lower for women who were told they were at higher risk. Further, understanding of risk results was variable, and examination of cancer worry ratings according to understanding of results suggests that cancer worry may be particularly high for women who (erroneously) thought their result indicated that they were likely to develop breast cancer.

The general absence of effects on intentions to change any behaviour suggest that providing risk information alone is unlikely to result in changes in behaviour without additional support being provided.^18^ Nevertheless, the provision of risk information is a necessary part of identifying women at higher risk who can then be offered additional screening or chemotherapy.

Given these results, progressing to further consider the feasibility of risk-stratified screening appears warranted (see also further evidence^23^). Increasing frequency of screening of women at high-risk and decreasing frequency of screening of women at low-risk is likely to lead to a better balance of benefits and harms of screening, and may be cost-neutral.

### Implications for research

The present research is currently the best available evidence on the likely harms and benefits of risk-stratified breast screening as part of the NHS Breast Screening Programme. Future studies of the feasibility of risk-stratified screening appear warranted, but could benefit from several improvements on the present research. First, future research should compare women who are offered personalised testing with women offered routine breast cancer screening, which would be a better comparator than women awaiting personalised test results. Allocation of women to these conditions should be done via randomisation or other procedures to ensure well-matched samples.

Future research should consider effects in both the short-term and longer-term. Such research should make greater use of theories of risk communication to better understand reactions to the information provided. It would be useful to examine effects on behaviour using validated tools, rather than intentions to change behaviour as in the present research. This could include objectively assessed behaviours such as attendance at future screening rounds and uptake of chemoprevention as well as changes in behaviour such as physical activity. Such research could examine how effects of risk communication on positive health behaviour could be facilitated. Future research could consider aspects of informed decision making other than just knowledge/ understanding of test results.

Should such work show there to be no major harms of stratified screening in terms of emotional and behavioural outcomes, and if uptake of the offer of stratified screening is sufficiently high, a full evaluation of the effectiveness and cost-effectiveness of such a programme would be warranted.

## Conclusions

The present research indicates that there would be no major emotional or behavioural harms of providing women with 10-year breast cancer risk estimates. Given this, further work of better quality to establish the feasibility of risk-stratified screening is warranted. As part of such work, there is a need to improve the risk communication materials to increase understanding and satisfaction with information, particularly for women at higher risk.

## Availability of data and materials

We are happy to share data and materials with bone fide researchers who provide a justification in writing for accessing this information.
